# Neutrophil Isolation Protocol

**DOI:** 10.3791/745

**Published:** 2008-07-23

**Authors:** Hana Oh, Brian Siano, Scott Diamond

**Affiliations:** Institute for Medicine and Engineering, University of Pennsylvania-School of Medicine

## Abstract

Neutrophil polymorphonuclear granulocytes (PMN) are the most abundant leukocytes in humans and among the first cells to arrive on the site of inflammatory immune response. Due to their key role in inflammation, neutrophil functions such as locomotion, cytokine production, phagocytosis, and tumor cell combat are extensively studied. To characterize the specific functions of neutrophils, a clean, fast, and reliable method of separating them from other blood cells is desirable for in vitro studies, especially since neutrophils are short-lived and should be used within 2-4 hours of collection. Here, we demonstrate a standard density gradient separation method to isolate human neutrophils from whole blood using commercially available separation media that is a mixture of sodium metrizoate and Dextran 500. The procedure consists of layering whole blood over the density gradient medium, centrifugation, separation of neutrophil layer, and lysis of residual erythrocytes. Cells are then washed, counted, and resuspended in buffer to desired concentration. When performed correctly, this method has been shown to yield samples of >95% neutrophils with >95% viability.

**Figure:**
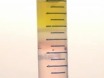


## Protocol

###  Neutrophil Isolation Protocol

1. Bring all reagents to room temperature.
2. Collect 5.0 ml of neutrophil isolation media in centrifuge tube. Carefully layer 5.0 ml of blood over the separation media. Perform this step slowly and carefully, and with the pipette tip close to the surface of the media to avoid mixing the blood and the media.
3. Centrifuge at 500 RCF for 35 min at 20-25°C. The blood should separate out into 6 distinct bands: plasma, monocytes, isolation media, neutrophils, more isolation media, and the red blood cell pellet (Figure 1). If these bands are not clear, the separation process was not clean and will need to be repeated.
4. Carefully remove the top three layers (plasma, monocytes, and isolation media) using a pipette. Dispose of these layers.
5. Carefully pipette the layer of neutrophils and all of the isolation media beneath the neutrophils. Place the solution into a clean centrifuge tube.
6. Dilute the neutrophil solution to 10 ml with HBSS without Ca^2^+/Mg^2^+. Invert the tube a few times to suspend the cells.
7. Centrifuge the neutrophil solution at 350 RCF for 10 minutes. A red pellet should be present at the bottom of the tube, containing neutrophils and residual red blood cells (RBCs). Remove the supernatant with a pipette carefully so that the pellet is not disturbed.
8. To lyse the residual RBCs, add 2 ml Red Cell Lysis Buffer to the tube. To resuspend the pellet, vortex the vial at a setting of 3-4. Avoid increasing the vortex setting above 4, since this may cause the neutrophils to activate. It may be necessary to vortex for several seconds, or to "pulse" the vortex to dissolve the pellet.
9. Centrifuge the tube at 250 RCF for 5 min. Remove the supernatant with pipette. Repeat the lysing process if required.
10. Add 500 μl HBSS without Ca^2^+/Mg^2^+ to each tube. Again, vortex to resuspend the pellet at a setting of 3-4. Dilute to 10 ml with HBSS without Ca^2^+/Mg^2^+.
11. Centrifuge the tubes at 250 RCF for 5 min. Aspirate the supernatant and discard.
12. Resuspend the pellet in 250 μl HBSS/HSA Solution (2% HSA). Cells may then be counted and adjusted to desired concentration.

 

## Discussion

The density gradient separation method is used to isolate human neutrophils from whole blood using a mixture of sodium metrizoate and Dextran 500. This method is based on the mononuclear leukocyte separation method by Boyum (1968) which was modified for neutrophil separation by Ferrante and Thong (1980). 

After collection from a donor, whole blood may be anticoagulated with EDTA, citrate, or heparin. Since they are short-lived, neutrophils should be used within 2-4 hours of collection. The procedure consists of layering whole blood over the density gradient medium, centrifugation, separation of neutrophil layer, and lysis of residual erythrocytes. Cells are then washed, counted, and resuspended to desired concentration. 

If the six bands are not distinct after the first centrifugation step, the separation process was not clean and will need to be repeated. For clean separation, make sure that the isolation media has not expired or contaminated. Separation may also be unsuccessful if the blood donor has consumed alcohol or medications in the 72 hours prior to blood collection. 

To prevent neutrophil activation during the separation procedure, it is best to use HBSS without Ca2+/Mg2+, since the ions have been shown to prime cells. Resuspension of pellets should also be performed slowly, and vortex settings should be kept in the low- to mid-range so that cells do not become activated. 

When performed correctly, this method has been shown to yield samples of >95% neutrophils with >95% viability. Purity and viability can be assessed by labeling the cells with neutrophil-specific marker CD66b and trypan blue dye exclusion.
